# *HE4* Transcription- and Splice Variants-Specific Expression in Endometrial Cancer and Correlation with Patient Survival

**DOI:** 10.3390/ijms141122655

**Published:** 2013-11-18

**Authors:** Shi-Wen Jiang, Haibin Chen, Sean Dowdy, Alex Fu, John Attewell, Eleftheria Kalogera, Ronny Drapkin, Karl Podratz, Russell Broaddus, Jinping Li

**Affiliations:** 1 Department of Biomedical Science, School of Medicine, Mercer University, Savannah, GA 31404, USA; E-Mails: jiang_s@mercer.edu (S.-W.J.); alex.a.fu@gmail.com (A.F.); 2 Division of Gynecologic Surgery, Mayo Clinic, Rochester, MN 55902, USA; E-Mails: dowdy.sean@mayo.edu (S.D.); kalogera.eleftheria@mayo.edu (E.K.); podratz.karl@mayo.edu (K.P.); 3 Department of Laboratory Oncology Research, Curtis and Elizabeth Anderson Cancer Institute, Memorial University Medical Center, Savannah, GA 31404, USA; 4 Department of Histology and Embryology, Shantou University Medical College, Shantou 515041, Guangdong, China; E-Mail: chenhb@stu.edu.cn; 5 Healthcare Analytics, Mayo Clinic, Rochester, MN 55902, USA; E-Mail: john.attewell@charter.net; 6 Department of Pathology, Harvard Medical School, Boston, MA 02115, USA; E-Mail: ronny_drapkin@dfci.harvard.edu; 7 Department of Pathology, the University of Texas MD Anderson Cancer Center, Houston, TX 77030, USA; E-Mail: rbroaddus@mdanderson.org

**Keywords:** *HE4*, transcription, splice variants, gene expression, endometrial cancer

## Abstract

We investigated the *HE4* variant-specific expression patterns in various normal tissues as well as in normal and malignant endometrial tissues. The relationships between mRNA variants and age, body weight, or survival are analyzed. ICAT-labeled normal and endometrial cancer (EC) tissues were analyzed with multidimensional liquid chromatography followed by tandem mass spectrometry. Levels of HE4 mRNA variants were measured by real-time PCR. Mean mRNA levels were compared among 16 normal endometrial samples, 14 grade 1 and 14 grade 3 endometrioid EC, 15 papillary serous EC, and 14 normal human tissue samples. The relationship between levels of *HE4* variants and EC patient characteristics was analyzed with the use of Pearson correlation test. We found that, although all five HE4 mRNA variants are detectable in normal tissue samples, their expression is highly tissue-specific, with epididymis, trachea, breast and endometrium containing the highest levels. *HE4*-V0, -V1, and -V3 are the most abundant variants in both normal and malignant tissues. All variants are significantly increased in both endometrioid and papillary serous EC, with higher levels observed in grade 3 endometrioid EC. In the EC group, *HE4*-V1, -V3, and -V4 levels inversely correlate with EC patient survival, whereas *HE4*-V0 levels positively correlate with age. *HE4* variants exhibit tissue-specific expression, suggesting that each variant may exert distinct functions in normal and malignant cells. *HE4* levels appear to correlate with EC patient survival in a variant-specific manner. When using HE4 as a biomarker for EC management, the effects of age should be considered.

## 1. Introduction

Endometrial cancer (EC) is the most common gynecologic malignancy and the fourth most common cancer in females, ranking behind breast, lung and colorectal cancers in the United States. While early stage EC has a favorable prognosis, late stage EC is associated with a relatively high rate of post-treatment recurrence and mortality. The importance of early diagnosis is underscored by the dramatic reduction of five-year survival rates from 96% to 67% and further to 17% for localized, regional, and metastatic disease, respectively [1]. Several studies have evaluated the efficacy of various tumor markers, including CA125, for EC early detection and management, but only limited specificities and sensitivities was observed [2–4].

HE4 was first cloned as one of four proteins highly expressed in human epididymis [5]. Amino acid sequence analysis suggested that HE4 belongs to a whey-acidic-protein (WAP) family and contains two four-disulfide core domains (WFDC2). *HE4* gene is located on chromosome 20q13.12, a region harboring a cluster of genes for WAP domain proteins [6–8]. Although it is well known that at least some members of this family serve as endogenous peptide protease inhibitors, the function of HE4 remains obscure. Using the cDNA microarray technique Schummer *et al.* found that *HE4* is overexpressed in ovarian cancer (OC) [9]. Bingle *et al.* used Northern blot hybridization in order to characterize its tissue-specific expression and found that HE4 mRNA is abundant in lung, kidney, and the salivary gland [8]. Lu *et al.* performed oligo-array analysis and observed that *HE4* is overexpressed in OC in comparison to normal ovarian epithelium [10]. Drs. Drapkin and Galgano *et al.* analyzed the expression of *HE4* in a series of normal and malignant tissues and observed high levels of HE4 mRNA in the trachea and salivary gland [11,12]. Tissue microarray studies indicate that HE4 protein is present in both normal and malignant ovarian and endometrial cells [10–13].

Bingle *et al.* compared the sequences of two lung-derived *HE4* ESTs (expressed sequence tags) with those of the prototypic HE4 cDNA, leading to the identification of five distinct HE4 mRNA variants [8]. Four of these variants (*HE4*-V0, -V1, -V2, -V4) are generated by differential splicing mechanisms whereas one (*HE4*-V3) utilizes an alternative promoter as suggested by the presence of an extended 5′ prime region. The disparate structures and domain arrangements among the *HE4* variants raised questions on their tissue-specific expression and potentially distinct cellular functions.

Being a small, secretory glycoprotein [11], HE4 is readily detectable in plasma and the serum levels of HE4 are considered a potential biomarker for ovarian cancer diagnosis and management [13–16]. An antibody-based ELISA has been developed to measure HE4 serum levels in various clinical settings [17–19]. It has been reported that HE4 alone or in combination with CA125 has higher sensitivity than any other single marker or combination of markers, in early detection [20–22], diagnosis [15,23–26], risk stratification [27] and prediction of postsurgical outcome [10,20,24,28] for patients with OC. With regards to EC, Moore *et al.* found that HE4 serum levels are consistently elevated in all stages of EC and that it is more sensitive in detecting early-stage disease than CA125 [29]. Bignotti *et al.* assessed the diagnostic and prognostic value of HE4 in EC. In their study, a significant correlation was found between increased HE4 serum levels and aggressive EC phenotype [30]. Based on these findings, HE4 test has been proposed as a valuable tool for the triage of patients presenting with a pelvic mass [31–33]. It is noteworthy that, despite the data in favor of HE4 protein levels as a useful biomarker, negative results from several studies challenged the predictive value of the ROMA algorithm when HE4 was used alone or in combination with CA125 [34,35].

While most studies suggest that HE4 is a clinically useful biomarker in EC and OC, the assays used to measure mRNA or protein levels were designed based on the structure of the prototype HE4, designated as HE4*-*V0 in this report. In previous studies, no distinction was made among the multiple structurally and potentially functionally diverged HE4 variants. The goal of this study is to compare HE4 mRNA variants-specific expression in normal and malignant endometrial tissues, and to analyze the relationship between HE4 variant-specific mRNA levels and clinical features including age, surgical stage and survival.

## 2. Results

### 2.1. HE4 Protein Is Overexpressed in Endometrial Cancer

Six pairs of matched normal and endometrial cancer tissue samples were prepared and analyzed on mass spectrometry. Figure S1 shows representative chromatograms for LC and mass spectrometry identification and comparison of HE4 levels between EC and normal endometrium samples. Thousands of proteins were identified from each of these pairs (3006, 2826, 2434, 2389, 1925, and 1803 for pairs 1 to 6, respectively; 75% confidence level). A more in-depth analysis of the data resulted in the identification of a group of 77 proteins which presented a greater than 1.5-fold changes in their expression levels between normal and cancer tissue in at least two out of six pairs of samples [36]. These proteins were found to be involved in a broad spectrum of cell functions including cellular structure determination, cell signaling, transcription control, as well as cell cycle and apoptosis regulation (Figure S1). Table 1 presents data on 8 representative proteins out of the 77 that exhibiting altered expression in endometrioid cancers. In accordance with previous studies [24,30], HE4 was found to be one of the 27 proteins significantly up-regulated in EC (Figure S2).

### 2.2. Design of HE4 Variant-Specific PCR Primers

**Figure 1:**
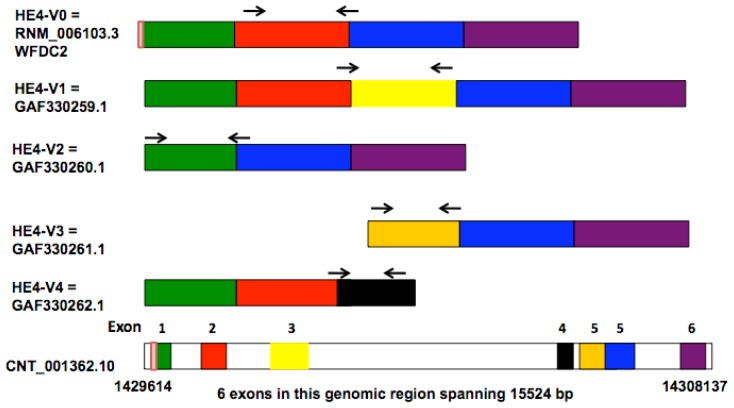
Schematic outline of real-time PCR primer design for each *HE4* variant. The structures of *HE4* variants were illustrated with the six exons marked in different colors. The locations of real-time PCR primers were indicated by arrows. On the bottom the *HE4* genomic structure of *HE4* prototype (*HE4*-V0) (NM_006103.3, GeneID: 10406) was shown based on the information from NCBI website [37]. The *HE4* gene is located at 20q13.12, spanning 1429614nt to 14308137nt in NM_006103.3.

**Figure 2:**
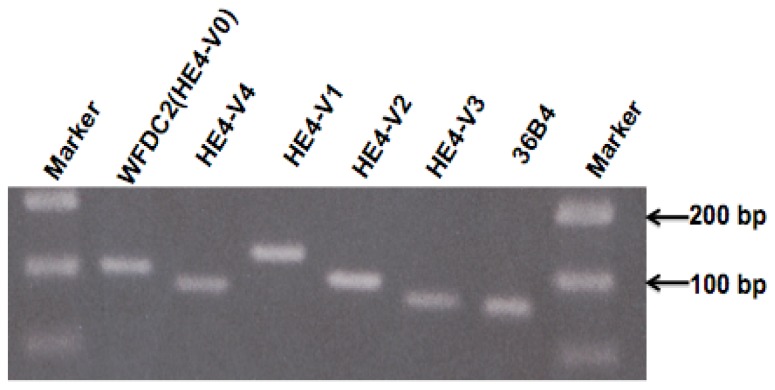
Gel electrophoresis of real-time PCR products. Real-time PCR products of the five *HE4* transcript variants are obtained from normal endometrial specimen, and separated in 2% agarose gel. The real-time PCR product size for each variant was: for *HE4*-V0, 112 bp; *HE4*-V1,131 bp; *HE4*-V2 96 bp; *HE4*-V3, 74 bp; *HE4*-V4, 84 bp. *36B4* (63 bp) was used as an internal control. The single band pattern indicates specific amplification of cDNA by real-time PCR.

We took advantage of the distinct intra-exon sequences and exon-exon boundaries within the *HE4* variants to design variant-specific PCR primers (Figure 1). The designations and sequences of these PCR primers are listed in Table 2. PCR conditions were extensively optimized to ensure specific and linear amplification. Aliquots of the real-time PCR products were resolved electrophoretically in agarose gels and subsequently visualized with ethidium bromide staining. A single band pattern with a predicted size indicated that specific amplification had been achieved (Figure 2).

### 2.3. Expression of HE4 mRNA Variants in Normal Tissues

**Figure 3:**
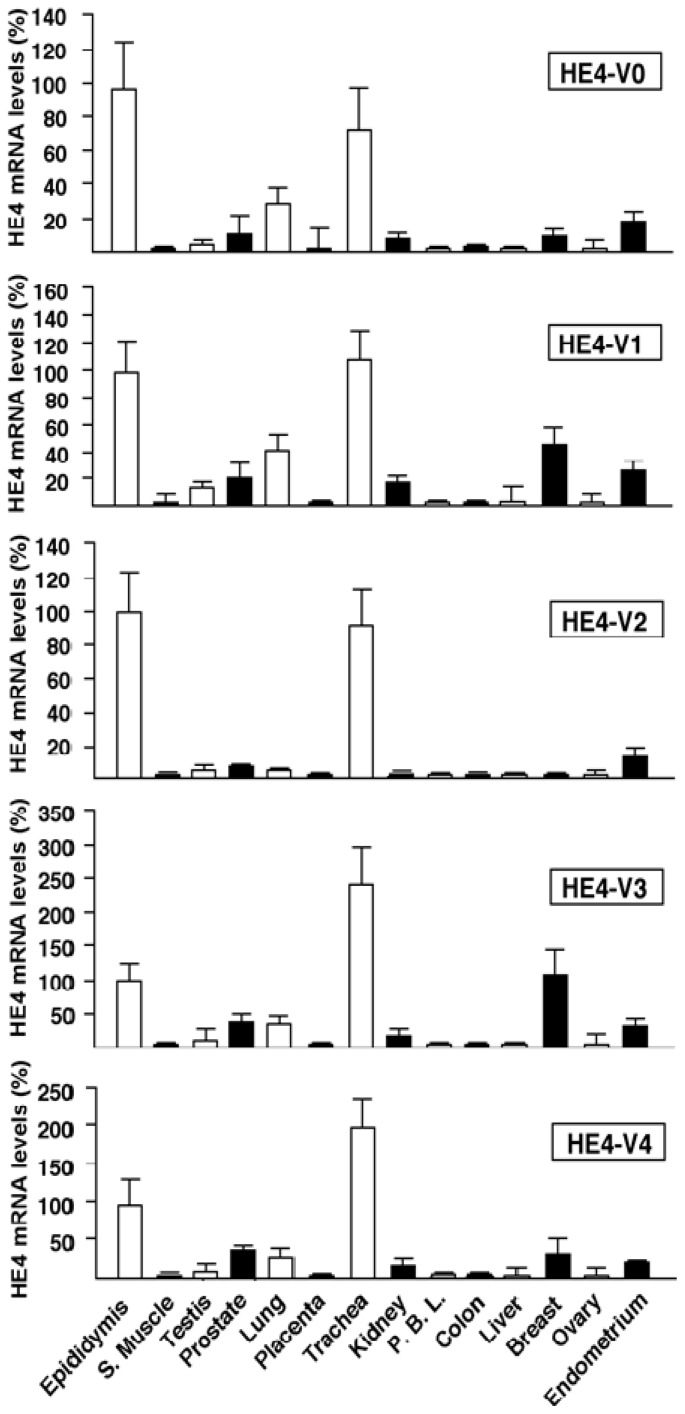
*HE4* variant expression levels in normal tissues. Total RNA was extracted from 14 types of normal human tissues: epididymis, skeletal muscle (S. Muscle), testis, prostate, lung, placenta, trachea, kidney, peripheral blood lymphocyte (PBL), colon, liver, breast, ovary and endometrium. Reverse transcription and real-time PCR were performed on these samples. The expression levels of each variant in different tissues were normalized by the level of epididymis (set as 100). The mRNA levels of HE4 were ranked in three groups: <5% was ranked as low, 5%–50% was ranked as moderate, >50% was ranked as high. In general, all the variants were expressed at the highest levels in epididymis or trachea, moderate levels in prostate, lung, breast and endometrium, and very low levels in skeletal muscle, testis, placenta, kidney, PBL, colon and liver.

To obtain general knowledge on the HE4 mRNA variants distribution, we first compared by real-time PCR the levels of their expression in 14 normal human tissues, including epididymis, skeletal muscle (s. muscle), testis, prostate, lung, placenta, trachea, kidney, peripheral blood lymphocyte (PBL), colon, liver, breast, ovary and endometrium. Relative mRNA levels of HE4 variants are shown in Figure 3, which indicates a high degree of variability across different tissues. Overall, epididymis and trachea express the highest levels of HE4 mRNA variants followed by breast, endometrium, lung and prostate, which expressed intermediate levels. Interestingly, this is consistent with Dr. Drapkin *et al.*’s previous finding [11]. The remaining tissues tested express only low to moderate levels of HE4 mRNA variants. Different tissues express diverged levels of *HE4* variants. For example, the highest levels of *HE4*-V0 and -V2 were found in the epididymis, whereas the highest levels of *HE4*-V1, -V3 and -V4 were in the trachea. *HE4*-V3 is also expressed at a relatively high level in breast tissue.

**Figure 4:**
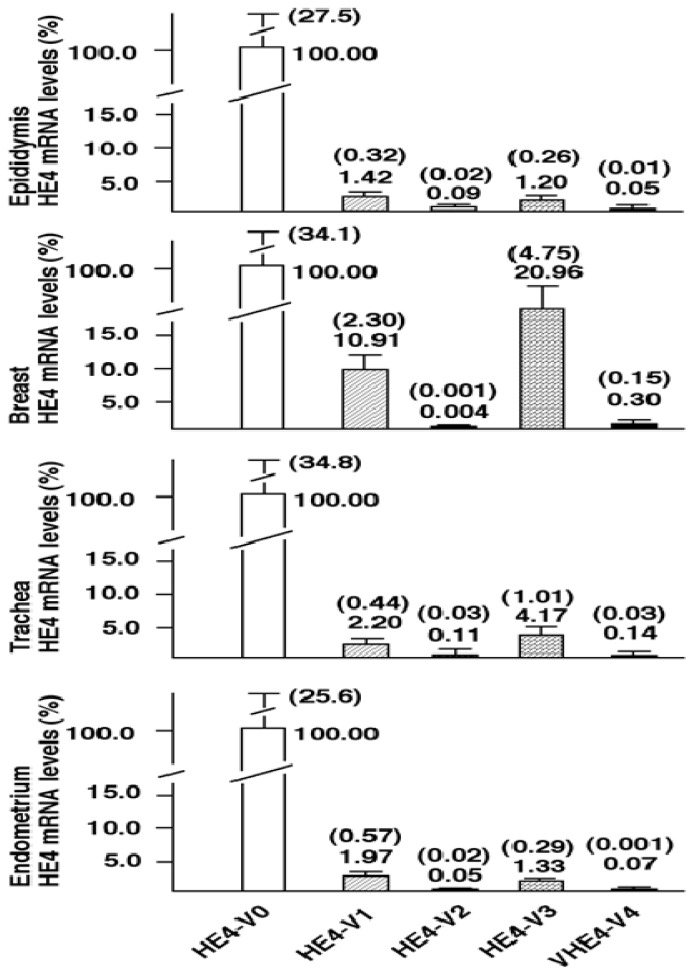
Comparison of *HE4* variant expression levels in normal tissues. The data from Figure 3 was re-set to best describe the *HE4* variant expression levels in epididymis, breast, trachea and endometrium. The relative expression levels were converted based on *HE4*-V0 (set as 100). Average and standard errors were indicated by the number on the top of bars.

In order to compare horizontally the levels of each variant across various tissues, the data was rearranged and presented by tissues. Figure 4 shows that the *HE4*-V0 to -V4 distribution in normal endometrium, breast, epididymis, and trachea. The distribution in the rest 10 types of tissues was shown in the supplemental data Figure S3. *HE4*-V0 represents the most abundant variant, followed by *HE4*-V1 and -V3 at approximately 10- to 100-fold lower levels than *HE4*-V0. Diminished levels of *HE4*-V2 and -V4, at approximately 100- to 1000-fold lower than *HE4*-V0, were detected in all the tissues.

To further analyze the tissue/organ-specific expression patterns of the 5 *HE4* variants, we performed regression analysis in the 13 normal tissues/organs. Similar expression patterns of *HE4* variants were found among the majority of the tissue types, with correlation coefficients all exceeding 0.88 and *p* values small than 0.05 (Table 3). Thus, the results indicated a significant inter-correlation among the different *HE4* variants (*N* = 5) in those normal tissues. Interestingly, no significant correlation in the *HE4* variant expression patterns could be established between the colon and several tissues such as ovary, PBL, placenta, kidney and prostate. Consequently, the colon appears to represent a special tissue with a unique *HE4* variant expression pattern when compared to other human tissues. It is intriguing to observe this highly consistent *HE4* variant expression patterns among a variety of human tissues, and at the same time, a dramatic divergence between colon and other tissues. The regulatory mechanism and functional significance of this phenomenon is not clear at this time.

### 2.4. Increased Levels of HE4 mRNA Variants in Endometrial Cancer

**Figure 5:**
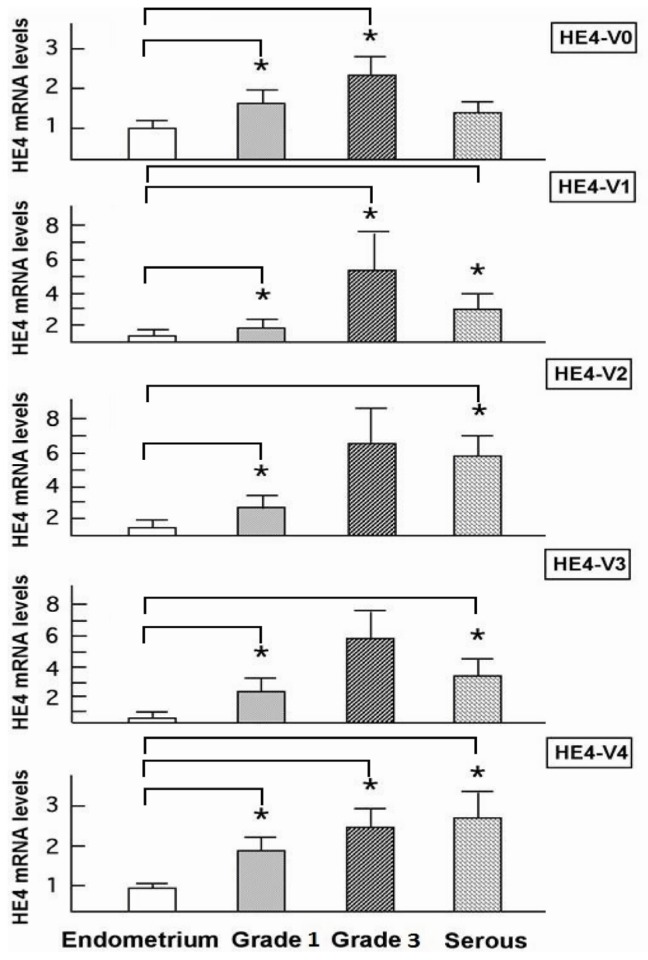
Increased *HE4* variant expression levels in EC. mRNA levels of the five HE4 variants were measured in normal endometrium, grade 1 and 3 endometrioid cancer, and serous cancers by real-time PCR. HE4 mRNA levels in normal endometrium were set as 1. Statistical significance (*p* < 0.05) was indicated by asterisks.

Using the same set of primers we performed real-time PCR to compare the *HE4* variant-specific expression between EC and normal endometrium tissues (Figure 5). The mRNA levels of HE4-V0, -V1, and -V4 were significantly higher in grade 1 and grade 3 endometrioid EC than in normal endometrium (*p* < 0.05). mRNA of HE4-V2 and -V3 were increased in grade 1 compared to normal endometrium (*p* < 0.05). The difference in the expression levels of *HE4*-V2 and -V3 in grade 3 EC compared to normal endometrium were of borderline significance (*p* = 0.057, *p* = 0.067, respectively). Likewise, the levels of all variants but *HE4*-V0 (*p* = 0.17) were significantly increased in papillary serous EC compared to normal endometrium (*p* < 0.05). The individual expression levels of each *HE4* variant compared to housekeeping gene measured in each clinical case were shown in the Figure S4 (supplemental data section). As it was observed in all normal tissues, *HE4*-V0 is the most abundant variant among the five counterparts in malignant as well as benign endometrial tissues.

While *HE4* expression patterns in grade 1 endometrioid, grade 3 endometrioid and papillary serous EC were significantly inter-correlated with each other (*p* < 0.05) (Table S1), they were not statistically correlated with the expression patterns in normal endometrium (*p =* 0.32, *p =* 0.18, *p =* 0.23 respectively), drawing a clear line that separate the malignant and normal endometrial tissues as two distinct entities.

### 2.5. Increased HE4 Expression Levels Are Negatively Associated with Patient Outcome

**Figure 6:**
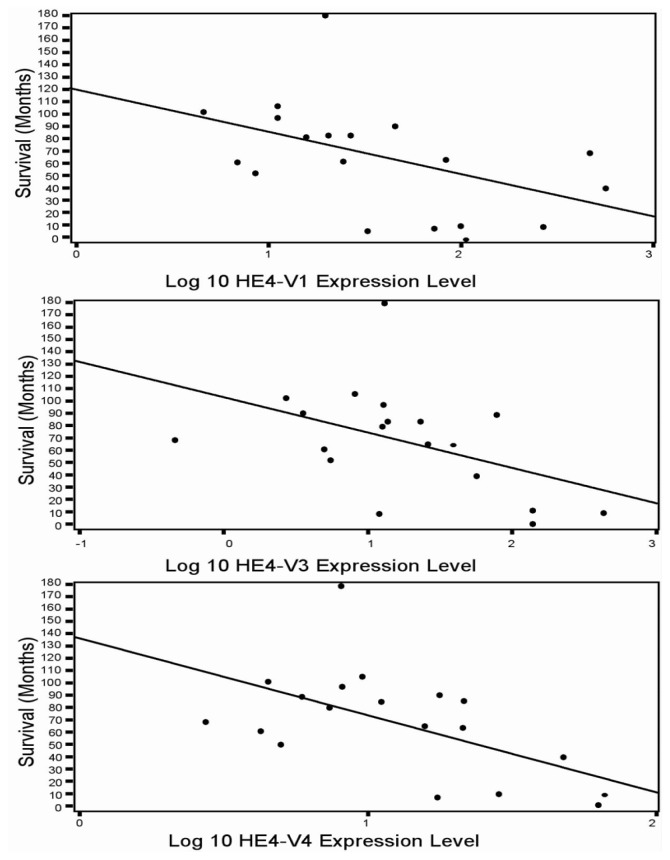
Correlation between HE4 variants mRNA levels and EC patient survival time. A significant inverse correlation was found between HE4-V1, -V3, -V4 mRNA levels and EC patient survival time in this endometrioid cancer sub-cohort (*p* = 0.031, *p* = 0.048, *p* = 0.01, respectively, *n* = 19).

The relationship between *HE4* variant expression levels and clinical outcomes were analyzed. Clinical data on survival, BMI and age are retrieved and summarized in supplemental Table S2. No statistically significant correlation was observed between *HE4* variants levels and age or BMI in the normal control group (Table 4). In contrast, a significant inverse correlation was found between HE4-V1, -V3, -V4 mRNA levels and EC patient survival time in grade 1 and grade 3 endometrioid cancer sub-cohort (Table 5) (*p* = 0.031, *p* = 0.048, *p* = 0.01, respectively). The detailed one-to-one correlation is documented in Figure 6. Interestingly, in the same endometrioid cancer sub-cohort, *HE4*-V0 levels were found to be positively correlated with patients’ age (*p* = 0.033), but not with body mass index (BMI) (*p* = 0.79) or survival (*p* = 0.23) (Table 5). Multivariate analysis adjusted by patient age and survival time confirmed that age was independently correlated with *HE4* levels. It is surprising to see a lack of significant correlation between mRNA levels of the five variants of HE4 and patient survival, BMI or age in the serous cancer group (Table 6). The mean levels of Age, BMI, and number of study subjects are presented in Table 7. The different relationships between *HE4* variant expression levels and clinical parameters in the endometrioid and serous tumors underscore a divergence between the type I and II EC cases, as expected by their differing etiology and molecular make-up.

## 3. Discussion

Utilization of alternative splicing or alternative promoters allows a single gene to encode multiple structurally and functionally related proteins. These processes have greatly increased the diversity of proteome, and may constitute an effective and economical mechanism for fine-tuned regulations of cell functions. Indeed, complex posttranscriptional regulatory mechanisms appear to be a common feature of the WAP domain genes. *Elafin* and *SLPI* genes, the two best-studied WAP domain family members, are both able to generate multiple splicing products [38–41]. Eppin, another recently identified WAP protein, is encoded by a single-copy gene as evidenced by the presence of single band on the Southern blotting of human genomic DNA [42]. Computer-aided analysis of *Eppin* sequence predicts the existence of three splice variants, all of which conform to the AG/GT splicing rule [42].

*HE4* gene generates five mRNA variants as a result of alternative splicing and utilization of alternative promoters [8]. Amino acid sequence and domain analysis of the deduced peptides predicts that the *HE4 N*-terminal and *C*-terminal WAP domains are encoded by exon 2 and exon 5, respectively. Based on the arrangement of WAP domains, the five *HE4* transcript variants can be categorized into three groups: Group 1 (*HE4*-V0 and -V1) contains both the *N*-terminal and *C*-terminal WAP domains; Group 2 (*HE4*-V4) contains only the *N*-terminal WAP domain; Group 3 *(HE4*-V2 and -V3) contains only the C-terminal WAP domain. In addition, *HE4*-V0, -V1, and -V4 share the same secretory signal peptide and are therefore likely to be present in the plasma. For the rest of *HE4* variants, HE4-V2 signal peptide is homologous to that of HE4-V0 with the exception of a deletion in the last 10 amino acids, suggesting differential regulation of secretion; HE4-V3 does not contain a consensus signal sequence, raising questions regarding its cellular location and secretory potential [8]. In this study, we provide experimental data showing that these structurally unique *HE4* variants are concurrently expressed in various human tissues. Although all variants were readily detectable by real-time PCR, their expression levels differ dramatically, by as many as 10,000 fold. Taken together, these data suggest that the production of *HE4* variants, either through differential splicing or use of alternative promoters, are subject to tight and distinct regulation. Furthermore, the structural divergence, differential locations, e.g., secreted form verses intracellular form, and markedly different expression patterns suggest that they may be implicated in tissue-specific functions.

Although total HE4 expressions have been characterized in a variety of normal and cancerous tissues by many laboratories [8,11,12], *HE4* variant-specific expression patterns has not been comprehensively investigated. We showed that the expression patterns of the five *HE4* variants share certain similarity among normal tissues. On the contrary, *HE4* variant expression patterns diverge significantly between malignant and normal endometrial tissues. This observation in conjunction with the fact that these variants are significantly overexpressed in endometrial cancer cells points to a potential role(s) for HE4 in EC tumorigenesis or progression. In a parallel study, we have tested this hypothesis. We demonstrated that at least the prototype HE4-V0 exhibits a potent tumor-promoting activity both *in vitro* and *in vivo* [43]. Given the fact that the deduced peptides from these mRNA variants contain a different number of WAP domains, and that the *N*- and *C*-terminal WAP domains share low homology, it is unlikely that these peptides carry out the same functions. Comparative variant-specific functional studies are required to delineate the roles of each variant in EC development.

In addition to its potential pathological role(s), HE4 levels may also reflect the status of EC progression from a clinical point of view. Yamashita *et al.* performed immunohistochemistry on lung adenocarcinoma tissue samples to investigate the predictive value of HE4 for patient outcome and found that the five-year disease-free survival in the HE4-positive group (44.6%) was significantly lower than that in the HE4-negative group (82.3%, *p* = 0.001). Moreover, HE4 expression and the nodal status were noted to be independent prognostic factors for disease-free and overall survival [44]. Kamei *et al.* examined the HE4 expression in breast cancer tissues and confirmed that HE4 protein level was associated with lymph node invasion. Furthermore, the five-year survival in the HE4-positive group (58.6%) was significantly worse than that in the HE4-negative group (85.6%, *p* = 0.04) [45]. Bignotti, *et al.* found that high serum HE4 levels correlated with reduced survival in the poorly differentiated EC cohort [30]. A study from our group recently indicated a correlation between the high serum HE4 levels and high surgical stage, increased myometrial invasion, and large primary tumor diameters in EC patients [46]. In the current study although all five HE4 mRNA variants were significantly increased in malignant compared to normal endometrium, only the levels of three variants (*HE4*-V1, -V3, and -V4) were found to be correlated with the patient survival data. This finding suggests that not all *HE4* variants are predictive of patient outcomes, which underscores the importance of a variant-specific approach in the measurement of *HE4* levels. It is noteworthy that the variants associated with survival (*HE4*-V1, -V3, and -V4) are those expressed at the highest level in the trachea but not the ones (*HE4*-V0 and -V2) expressed at the highest levels in the epididymis. Neither the interpretation nor the significance of this observation is clear at this time.

Despite the ambiguity regarding each variant’s distinct function, correlation between the expression of certain *HE4* variants and patient survival may prove important from a clinical perspective. Numerous studies have shown that mRNA detection in serum as well as in other body fluids may provide a promising approach to cancer assessment and management. Tumor cell mRNA in plasma may originate from necrosis or apoptosis of circulating cancer cells, or as the result of an active release mechanism in which mRNA transportation occurs through vesicle-like structures [47,48]. Based on the high expression levels of the HE4 mRNA variants in EC tissue samples, the same mRNA variants may be detectable in patients’ serum as well. Thus, the variant-specific approach may offer insights into EC detection, prognostication, and disease monitoring. The optimal technical parameters we outline here could serve as a foundation for future studies along this direction.

Because both ovary and endometrium develop from the Müllerian system [49–51], carcinomas arising from these sites tend to have similar etiological factors, gene expression profiles, and tumorigenic mechanisms. Consequently, defining the expression patterns of *HE4* variants in normal and malignant ovarian tissues may demonstrate similar findings. However, early detection of *HE4* variants is more convenient in EC compared to ovarian or other types of cancer given that tumor cells can be collected non-invasively in menses or vaginal secretions by the use of pap smear-like devices or tampons [52]. RNA samples extracted from pap smear specimens have been successfully utilized for transcriptome profiling in the past [53]. The currently available HE4 ELISA assay measures the total HE4 levels and does not distinguish the HE4 protein species produced by different HE4 mRNA variants. One could envision using either antibody-based ELISA or mRNA-based PCR assays to detect HE4 mRNA variants in these body fluids for the purpose of early EC detection.

Anastasi *et al.* observed that serum HE4 levels vary substantially among the follicular, ovulatory and luteal phases in healthy young women, suggesting hormonal regulation of HE4 production in the ovary and other somatic tissues [28]; recently, Levanon *et al.* have also shown that normal fallopian tube secretes HE4, these data indicate the characteristic of the HE4 origin from the Mullerian epithelium [54]. Malignant endometrial cells express varied levels of steroid hormone receptors due to differential epigenetic modification. This may have a significant effect on HE4 variant expression patterns and at the same time, cancers’ response to hormonal therapy. Since most of the patients included in our study were postmenopausal women, we could not assess the influence of menstrual cycle on HE4 expression. Interestingly, *HE4*-V0, the most abundant *HE4* variant, was found to be correlated with age in the endometrioid cancer cohort. This result is consistent with another study showing that serum HE4 levels are closely correlated with age in EC patients [46]. Given the fact that the relationship between HE4 mRNA levels and age is not evident in the normal control group, this age-related change of *HE4* levels may reflect tumor characteristics rather than varying hormonal status. This hypothesis is further supported by the data from our recent study in which HE4 serum levels positively correlated with high-risk tumor characteristics in EC [46]. The association between *HE4*/HE4 levels and age also suggest that the age-adjusted HE4 levels may be more accurate criteria for cancer diagnosis and prognostication. We should point out that this observation is in agreement with the data recently published by Bolstad *et al.* [55]. In that study, it was found that higher HE4 levels are associated with age and smoking status, as well as creatinine levels and BMI in healthy subjects, while the two later factors are also associated with age. These new findings are very important in the way that when *HE4* variants are applied as a biomarker for EC diagnosis, these factors need to be taken into account to rule out the possibility that the elevated *HE4* levels may be caused by the patients’ age, smoking status and BMI in addition to the tumor itself. As the higher creatinine levels and smoking status are associated with age, the relationship between HE4 levels and creatinine levels would be an interesting issue for future studies.

This study is to our knowledge the first one to demonstrate a tissue-specific distribution of *HE4* variants and establish an *HE4* variant-specific correlation with survival in EC. Furthermore, we suggest that a variant-specific approach may prove useful for improving the current use of HE4 assays in the diagnosis, triage and postsurgical surveillance for the patient with EC.

## 4. Experimental Section

### 4.1. Collection of Tissue Samples

Both normal and malignant endometrial tissue samples were collected randomly from patients treated at Mayo Clinic for a variety of gynecologic conditions. Central pathology review was conducted to verify institutional diagnoses and eliminate variability among experts. Samples obtained from patients undergoing hysterectomy for benign indications were used as normal endometrium controls. Endometrial tissue specimens were obtained from 14 patients with grade 1 EC, 14 patients with grade 3 endometrioid EC, and 15 patients with papillary serous EC, and 16 patients with normal endometrium. For the six pairs of tissues analyzed by mass spectrometry study, EC and normal samples were selected based on match criteria of age (±5 years), body weight (±10 kg) and menopausal status, which is a separate pool of the above described cohort. Clinical data on survival, BMI and age were retrieved and confirmed for most of the patients. All samples were snap frozen, stored at −80 °C. For total RNA extraction, tissues were cut into 10 μm-thick series sections; one section was used for H&E staining to define the tumor tissues (circled with marker) *vs.* myometrium. 10 series sections were matched with H&E stained one, only tumor tissues were macro-dissected to ensure that at least 50% of the samples were tumor tissue. These studies were approved by the Institutional Review Board of Mayo Foundation (IRB# 07-004290). In accordance with the Helsinki Declaration and Minnesota Statute for Use of Medical Information in Research, only patients who consented to the use of their medical records were included in the study.

### 4.2. Tissue Preparation and ICAT Labeling Procedure

Specimens were mixed with 0.5 ml of lysis buffer (0.5 mM pH 8.3 Tris plus 0.1% SDS), and a glass grinder was used to homogenize the tissues. After separating the supernatant from the tissue debris by centrifugation at 13523 RCF (or 12,000 rpm), the supernatant was used for Isotope-Coded Affinity Tag (ICAT) labeling for a high throughput comparison of normal and malignant endometrial tissues. Protein concentrations were measured using the Bio-Rad Protein Assay Kit (Bio-Rad, Hercule, CA, USA). 100 μg of protein was labeled with Cleavable ICAT (cICAT) purchased from Applied Biosystems (Applied Biosystems, Foster City, CA, USA). We labeled the control samples with light isotopes (containing 8 hydrogens, D_0_) and the EC samples with heavy isotopes (containing 8 deuteriums, D_8_). Briefly, protein extracts were mixed with 2 μL of reducing agent. After boiling for 10 min, the labeling reaction was carried out overnight at 37 °C. Each labeled EC sample was combined with its labeled control sample and the mixture was incubated overnight at 37 °C in digestion buffer solution containing 2 μL of CaCl_2_ and 1 vial of trypsin as provided in the kit. Following digestion, the peptide mixture was subject to cation ion exchange purification and affinity purification using an Avidin column to collect peptides containing ICAT-labeled cysteine. 100 μL of cleavage reagent was added and the mixture was kept at 37 °C for 2 h to cleave the biotin portion. Cleavage mixture was dried under vacuum and stored at −80 °C until mass spectrometric analysis.

### 4.3. Multidimensional Liquid Chromatography Coupled to Tandem Mass Spectrometry (LC/MS/MS)

The cleaved samples were separated into 8 fractions on a cation exchange column (Biox SCX 300 mm × 5 cm, Dionex, Sunnyvale, CA, USA) using an off-line Agilent 1100 series capillary liquid chromatography system (Wilmington, DE, USA). The API QSTAR Pulsar I, a hybrid quadrupole time-of-flight mass spectrometer (Applied Biosystems, Foster City, CA, USA) was used to perform the LC/MS/MS analysis of the peptides from each fraction with the help of the Analyst QS 1.0 software (Applied Biosystems, Foster City, CA, USA). The QSTAR was configured with the Protana Nanospray Source (Proxeon, Denmark), and coupled to an Ultimate Nano liquid chromatography system (Dionex, Sunnyvale, CA, USA) equipped with a Zorbax C18 100 mm × 150 mm microbore column (Agilents, Wilmington, DE, USA). Mass spectrometry analysis consists of a one-second survey scan from 400 to 1600 mass-to-charge ratio (*m*/*z*) followed by two-second MS/MS fragmentation ion scan with a threshold of 10 counts per second. Once the three most intense ions were fragmented in each survey scan, they were excluded from repeated fragmentation for 60 s. The collision energy applied varied automatically depending on the precursor m/z and charge state. The peptide mass tolerance was set at 0.2 Da.

### 4.4. Data Analysis of LC/ICAT

Acquired data were searched against the CDS FASTA database and quantified using ProICAT 1.0 SP3 software (Applied Biosystems, Foster City, CA, USA). Relative quantification of proteins was performed on the TOF (time of flight) MS scan by calculating the ratio between the areas representing the light- and heavy-labeled peptide peaks. Two different search engines were used for protein identification, the ProICAT and the Mascot (Matrix Science, Boston, MA, USA) from the human non-redundant database. Only the proteins with a confidence level of 90% were used for further identification and quantification. The protein function annotation was supplemented using an online bioinformatics program, the Bioinformatics Harvester [56]. All proteins identified (Table 1) were individually verified by manually inspecting its precursor on chromatogram and mass spectrum. The protein accession numbers (GI number) are from NCBI protein database.

### 4.5. RNA Isolation, cDNA Synthesis, and Quantitative PCR

Total RNA of endometrial tissues was isolated from 20 μm-thick sections of frozen tissue using TRIzol^®^ reagent (Invitrogen, Carlsbad, CA, USA) followed the protocol described previously [57]. All the RNA samples were treated with DNase (RNase-Free DNase Set, cat No. 79254, Qiagen, Valencia, CA, USA) before reverse transcription. The purity of RNA has been determined by OD260/280 ratio. The quality of RNA and cDNA was ensured by the results of pilot PCR in which clear specific DNA band of the target gene was observed in agarose gel electrophoresis. Poly-A RNA from human epididymis, skeletal muscle (s. muscle), testis, prostate, lung, placenta, trachea, kidney, peripheral blood lymphocyte (PBL), colon, liver, breast, and ovary were purchased from Clontech Laboratories (Clontech Laboratories, Inc., Mountain View, CA, USA). cDNA was synthesized from 1 μg of total RNA using the SuperScript™ kit (Invitrogen, Carlsbad, CA, USA). 20 μL of reverse transcription product was diluted to 100 μL and 2 μL used for each real-time PCR reaction. The cDNA sequences for human *HE4* transcript variants (*HE4*-V0 to -V4) and human *36B4* (internal control) and *GAPDH* (internal control) were obtained from the PubMed gene bank and primers were designed by using primer 3 software [58]. All the primers were synthesized from IDT (Integrated DNA Technology). *36B4* is a ribosomal RNA and ubiquitously expressed in all the cells. Owing to its housekeeping function, and the fact that CT value of *36B4* is lower than the CT value of *GAPDH*, and *36B4* has lower variance, *36B4* was used as a control experiment for real-time PCR. We have also used *36B4* as housekeeping gene in previously works [59–61]. Negative control has been performed on the same 96-well PCR plate to test the PCR specificity. Real-time PCR was performed following previously described protocols [57,61] in a 25 μL reaction containing 2 μL of cDNA, 12.5 μL of SYBR^®^ Green PCR Master Mix (Stratagene, Cedar Creek, TX, USA) and 50 nM of forward and reverse primers, respectively. The ABI 7900HT Fast Real-time PCR^®^ System (Applied Biosystems, Foster City, CA, USA) was used with the following regimen of thermal cycling: Stage 1: 1 cycle, 2 min at 50 °C; Stage 2: 1 cycle, 5 min at 95 °C; Stage 3: 40 cycles, 15 s at 95 °C, 1 min at 60 °C.

Relative mRNA concentrations were calculated as the following: The threshold cycle number (CT) at which PCR products reached a preset threshold value, which was defined as the value where all products were undergoing exponential amplification, was determined by the florescence detector. Real-time PCR for the housekeeping gene *36B4* was performed in the same plates as for *HE4* experiments. The *HE4* real-time PCR results were standardized by those of *36B4* using the formula Δ*CT* = *CT_HE4_* − *CT_36B4_* (*CT_HE4_* and *CT_36B4_*, threshold values for *HE4* and *36B4*, respectively). Relative HE4 mRNA levels were expressed as fold over *36B4* mRNA levels (*F* = 2^Δ^*^CT^*). The experiments were repeated at least twice and the *CT* for each sample was determined in duplicate or triplicate. For practical reasons, relatively HE4 mRNA levels were arbitrarily amplified by a factor of 100 or 10,000 as indicated in figure legends. All the real-time PCR data were normalized with housekeeping gene.

### 4.6. Statistical Analysis

Mean values among different groups were compared (grade 1 or grade 3 endometrioid EC or papillary serous EC *versus* normal endometrium) using a *post-hoc* Bonferroni *t* test with the assumption that HE4 mRNA levels exhibited bimodal distribution and that the variances of the two samples were equal. Pearson’s correlation coefficient test was used to examine the relationship between the expression patterns of the five *HE4* variants among normal tissues as well as normal and malignant endometrium. Pearson’s correlation coefficient test was also applied to study the relationship between HE4 mRNA levels and patient characteristics including age, surgical stage, and disease free survival. To avoid overly influential observations, our data were log-transformed before statistical analysis. Correlations with a *p* value smaller than 0.05 and a coefficient close to 1.0 were considered statistically significant.

## 5. Conclusions

This study is to our knowledge the first one to demonstrate a tissue-specific distribution of *HE4* variants and establish an *HE4* variant-specific correlation with survival in EC. Furthermore, we suggest that a variant-specific approach may prove useful for improving the current use of HE4 assays in the diagnosis, triage and postsurgical surveillance for the patient with EC.

## Supplementary Information



## Figures and Tables

**Table 1:** Representative proteins identified by Isotope-Coded Affinity Tag (ICAT)-mass spectrometry. The table provides basic information on accession number (GI number is NCBI/PubMed protein ID number), protein identity, cellular function, numbers of paired-samples in which the protein was detected (Presence), the average ratio between endometrioid cancers and normal controls (Mean), and the standard errors (STE).

Accession ID number	Protein identity	Function	Presence	Peptide	Mean	STE
GI: 32307109	HE4, WAP four-disulfide core domain protein 3 precursor, WFDC2	Male fertility function. Putative ovarian tumor marker	2	1	2.45	1.23
GI: 3859849	LIM protein SLIMMER	Intracellular signaling pathway	3	1	2.39	1.11
GI: 15779184	FLNA protein, Filamin	Actin binding, cell structure and mobility	4	1	2.00	0.57
GI: 4504165	Gelsolin	Calcium-regulated, actin modulation; Silenced in tumor cells	2	2	0.52	0.07
GI: 86947	Galectin 1, beta-galactoside-binding lectin, placental	Cell differentiation and apoptosis	5	2	0.25	0.05
GI: 3193336	DBI-related protein	Fatty acid metabolism	2	1	0.54	0.20
GI: 37544577	Hypothetical protein XP_294867	Unknown	5	4	1.60	0.13
GI: 27545315	Translational activator of cytochrome c oxidase 1	Translational activator	5	10	1.58	0.21

**Table 2:** Real-time PCR primers for *HE4* transcript variants. The sequences and designation of PCR primers for real-time PCR measurement of *HE4* transcript variant were listed.

Primer name	Primer sequence
WFDC2 = *HE4-*V0 Forward	5′-CCG ACA ACC TCA AGT GCT G-3′
WFDC2 = *HE4-*V0 Reverse	5′-CGA GCT GGG GAA AGT TAA TG-3′
*HE4-*V1 Forward	5′-AAT GGC CAA CTG GCT GAG-3′
*HE4-*V1 Reverse	5′-TTT GAG AGA GTC CCC AGC TC-3′
*HE4-*V2 Forward	5′-CCA TGC CTG CTT GTC GCC-3′
*HE4-*V2 Reverse	5′-CAG GAA CCC TCC TTA TCT GA-3′
*HE4-*V3 Forward	5′-GCC ATG CTG CAG GTA CAA GT-3′
*HE4-*V3 Reverse	5′-ATC TGG GTA GAA AAA GGA GTA AGG-3′
*HE4-*V4 Forward	5′-CCC AAT GCA CTG TTC CAC T-3′
*HE4-*V4 Reverse	5′-AGT CCC AAG TGG GCC TTC-3′
*36B4* Forward	5′-ATG CAG CAG ATC CGC ATG-3′
*36B4* Reverse	5′-TCA TGG TGT TCT TGC CCA TCA-3′

**Table 3:** Correlation analysis of HE4 splicing variants among normal tissues. There was a significant correlation between the *HE4* variant expression patterns among the all the normal tissues except for Colon (*r* > 0.88, *p* < 0.05). The expression pattern in Colon was not associated with those of Ovary, Placenta, PBL, Prostate and Kidney (marked as ^*^).

	Colon	Endometrial	Epidydimus	Kidney	Liver	Lung	S. muscle	Ovary	PBL	Placental	Prostate	Testis	Trachea
colon	1	0.936	0.963	0.877	0.892	0.887	0.886	0.871	0.858	0.812	0.869	0.945	0.913
prob > |*r*|		0.019	0.009	0.051^*^	0.042	0.045	0.046	0.055 ^*^	0.063 ^*^	0.095 ^*^	0.056 ^*^	0.015	0.031
endometrial		1	0.993	0.987	0.991	0.991	0.975	0.986	0.980	0.944	0.985	0.994	0.991
prob > |*r*|			0.001	0.002	0.001	0.001	0.005	0.002	0.004	0.016	0.002	0.001	0.001
epidydimus			1	0.963	0.976	0.969	0.958	0.961	0.950	0.929	0.965	0.985	0.986
prob > |*r*|				0.008	0.004	0.007	0.010	0.009	0.014	0.022	0.008	0.002	0.002
kidney				1	0.995	1.000	0.963	1.000	0.995	0.962	0.997	0.985	0.984
prob > |*r*|					0.000	<0.0001	0.009	<0.0001	0.000	0.009	0.000	0.002	0.002
liver					1	0.995	0.955	0.994	0.984	0.977	0.999	0.984	0.996
prob > |*r*|						0.000	0.011	0.001	0.003	0.004	<0.0001	0.003	0.000
lung						1	0.970	0.999	0.996	0.957	0.996	0.987	0.985
prob > |*r*|							0.006	<0.0001	0.000	0.011	0.000	0.002	0.002
s. muscle							1	0.967	0.977	0.885	0.953	0.959	0.947
prob > |*r*|								0.007	0.004	0.046	0.012	0.010	0.015
ovary								1	0.997	0.960	0.996	0.981	0.982
prob > |*r*|									0.000	0.010	0.000	0.003	0.003
PBL									1	0.938	0.987	0.975	0.967
prob > |*r*|										0.019	0.002	0.005	0.007
placental										1	0.980	0.928	0.977
prob > |*r*|											0.004	0.023	0.004
prostate											1	0.977	0.991
prob > |*r*|												0.004	0.001
testis												1	0.981
prob > |*r*|													0.003
trachea													1
prob > |*r*|													

**Table 4:** Correlation of *HE4* variants expression levels with endometrial cancer (EC) patients’ clinical data. The Pearson correlation coefficient analysis is performed between *HE4* variant levels and patients’ age, BMI, and survival time. No significant correlation between *HE4* variant levels and clinical data was found in normal endometrium group (Table 4).

	BMI	Age
**Log_10_(V0)**	**−0.172**	**−0.184**
	*p* = 0.524	0.495
	*n* = 16	16
**Log_10_(V1)**	**0.086**	**−0.076**
	*p* = 0.752	0.779
	*n* = 16	16
**Log_10_(V2)**	**0.046**	**−0.075**
	*p* = 0.865	0.782
	*n* = 16	16
**Log_10_(V3)**	**−0.087**	**0.377**
	*p* = 0.749	0.150
	*n* = 16	16
**Log_10_(V4)**	**0.229**	**0.134**
	*p* = 0.394	0.620
	*n* = 16	16

Prob > |*r*| under H0:Rho = 0.

**Table 5:** Correlation of *HE4* variants expression levels with endometriod EC patients’ clinical data. A significant inverse correlation between three of the variants (*HE4*-V1, -V3, -V4) and patients’ survival were found in endometrioid EC group. There was also a positive correlation found between variants *HE4*-V0 and patients’ age. No significant correlation was found between *HE4* expression levels and patients’ BMI in this group.

	Survival	BMI	Age
**Log_10_(V0)**	**−0.285**	**−0.057**	**0.419**
	*p* = 0.236	0.791	0.033
	*n* = 19	24	26
**Log_10_(V1)**	**−0.497**	**−0.114**	**0.226**
	*p* = 0.031	0.595	0.267
	*n* = 19	24	26
**Log_10_(V2)**	**−0.338**	**−0.085**	**0.179**
	*p* = 0.157	0.692	0.380
	*n* = 19	24	26
**Log_10_(V3)**	**−0.459**	**−0.038**	**0.262**
	*p* = 0.048	0.858	0.196
	*n* = 19	24	26
**Log_10_(V4)**	**−0.572**	**−0.056**	**0.299**
	*p* = 0.011	0.796	0.138
	*n* = 19	24	26

Prob > |*r*| under H0:Rho = 0.

**Table 6:** Correlation of *HE4* variants expression levels with Serous EC patients’ clinical data. In serous cancer group, there was no significant correlation between the variants expression and patients’ survival, BMI and Age.

	Survival	BMI	Age
**Log_10_(V0)**	**0.088**	**0.253**	**−0.075**
	*p* = 0.774	0.364	0.790
	*n* = 13	15	15
**Log_10_(V1)**	**0.304**	**0.145**	**−0.077**
	*p* = 0.313	0.605	0.786
	*n* = 13	15	15
**Log_10_(V2)**	**0.247**	**0.454**	**−0.254**
	*p* = 0.416	0.089	0.361
	*n* = 13	15	15
**Log_10_(V3)**	**0.32**	**0.112**	**−0.085**
	*p* = 0.287	0.691	0.763
	*n* = 13	15	15
**Log_10_(V4)**	**0.332**	**0.482**	**−0.074**
	*p* = 0.268	0.069	0.794
	*n* = 13	15	15

Prob > |*r*| under H0:Rho = 0.

**Table 7:** In endometrioid cancer group, the cases numbers (*N*), mean value (mean), the maximum value and minimum value on patients’ survival, BMI and age are summarized.

	*N*	Mean	σ	Minimum	Maximum
Survival	19	68.7	43.1	1	180
BMI	24	34.4	9.4	19.6	63.5
Age	26	59.7	12.8	39	91
