# Supplementary material for: HE4 Transcription- and Splice Variants-Specific Expression in Endometrial Cancer and Correlation with Patient Survival
Source: Int J Mol Sci. 2013 Nov 18;14(11):22655–77. doi: 10.3390/ijms141122655 (PMC3856083; doi:10.3390/ijms141122655)
Supplement: Supplementary file 1 [file ijms-14-22655-s001.pdf]

Supplementary Information

Figure S1. Distribution of proteins differentially expressed in EC. Six pairs of EC and normal endometrial tissues are analyzed on mass spectrometry. The expression levels in 77 proteins were found to be altered with 1.5 or more fold of changes in EC compared to normal endometrium in at least 2 out of 6 pairs. As presented in the pie chart, these proteins are involved in diversified cell functions.

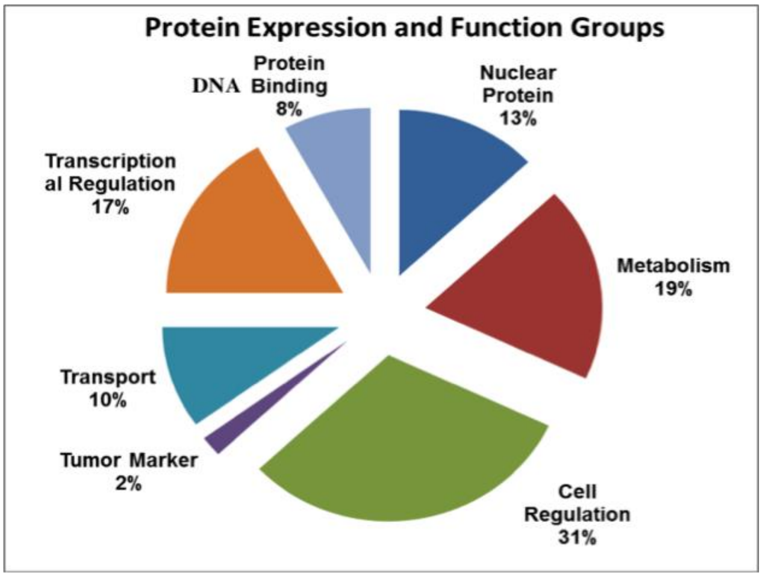

Figure S2. Proteomic identification and quantification of HE4. Upper panel: The base peak chromatogram of fractions containing HE4 peptide was shown at a retention time of 70 min. HE4-containing fragment from the above fraction was analyzed by tandem mass spectrometer. Lower panel: MS/MS spectrum of the precursor ion at 489.63 *m*/*z* identifies the heavy ICAT-labeled peptide (cancer patient) and at 486.6214 *m*/*z* identifies the light ICAT-labeled peptide (control). The ratio for HE4 changes in EC was obtained by comparing the areas under the curves of the two peaks.

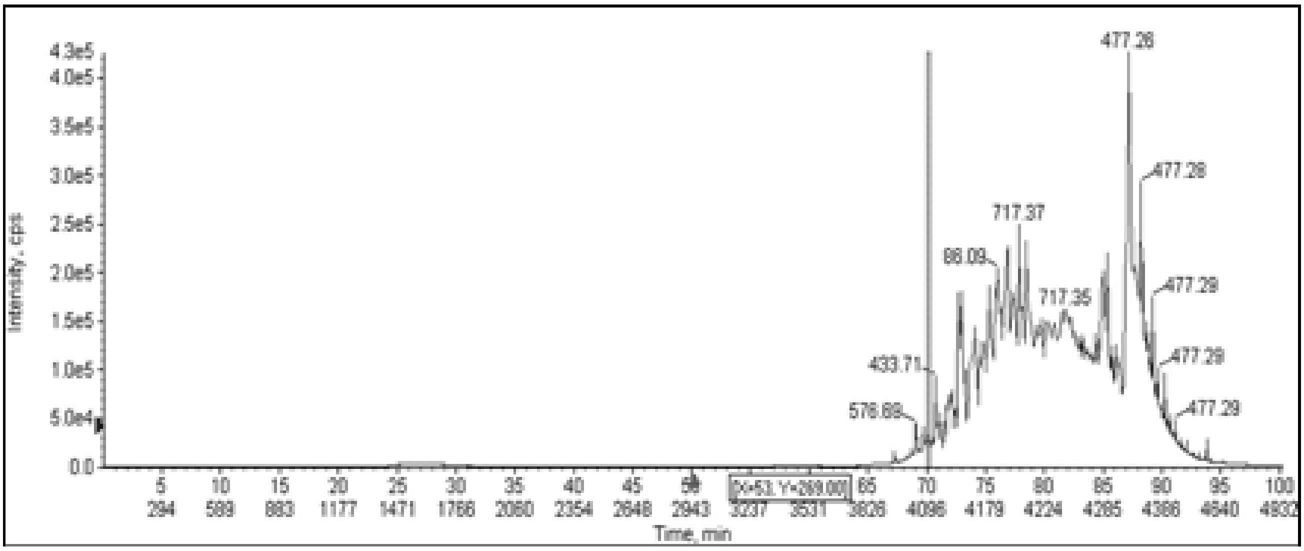

Figure S2. *Cont.*

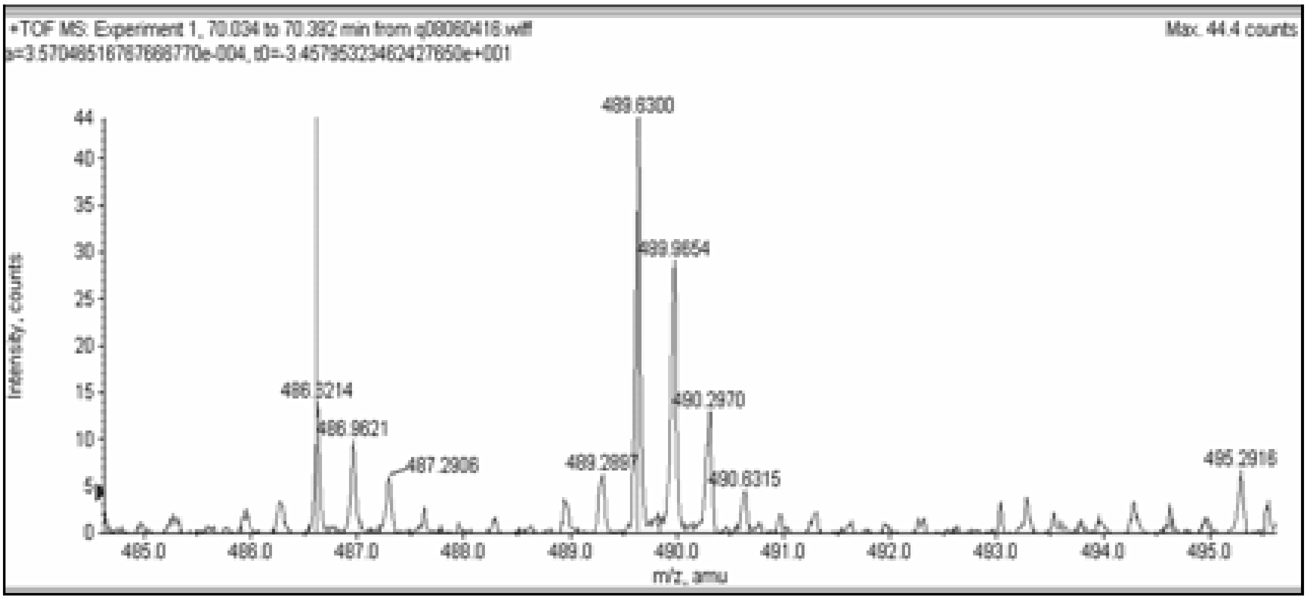

Figure S3. HE4 mRNA variants expression levels in 10 normal tissues. The mRNA levels of 5 HE4 variants are measured in 10 normal human tissues including epidydimus, skeletal muscle (S. muscle), testis, prostate, placenta, kidney, peripheral blood lymphocyte (PBL), colon, liver, and ovary by real-time PCR. The relative expression levels of each HE4 variant (HE4-V1 to -V4) are presented as the percentage of HE4-V0 (set as 100). The most abundant variant is HE4-V0 in all the 10 tissues. HE4-V3 is also expressed at relatively high level in the placenta.

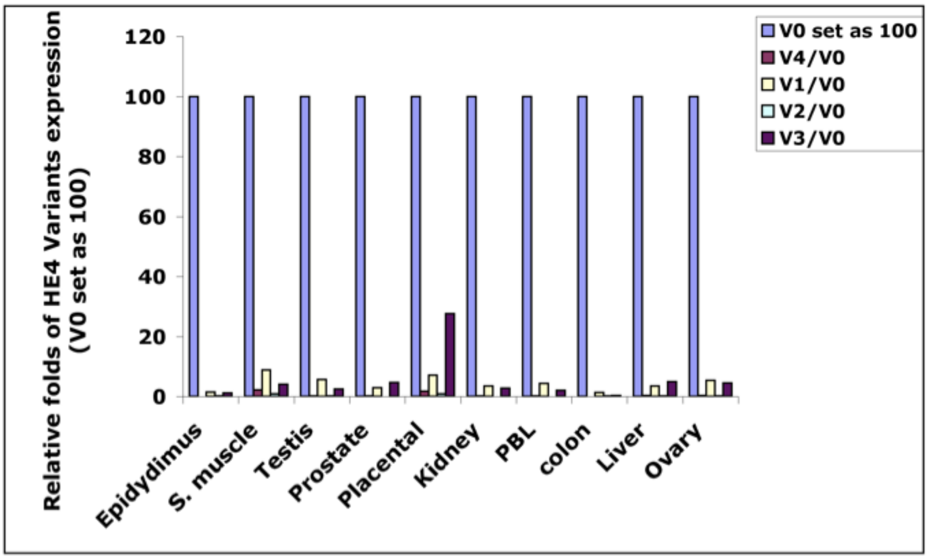

Figure S4. HE4 mRNA variants expression in EC and normal endometrium. Expression levels of the 5 HE4 mRNA variants are measured real-time PCR. Each chart shows the expression level of a specific variant in the 4 types of tissues: normal endometrium (1 to 16), Grade 1 endometrioid EC (17 to 30), in Grade 3 endometrioid EC (31 to 44), and papillary serous EC (45 to 59). Each bar represents the average of three parallel experiments on the same clinical specimen.

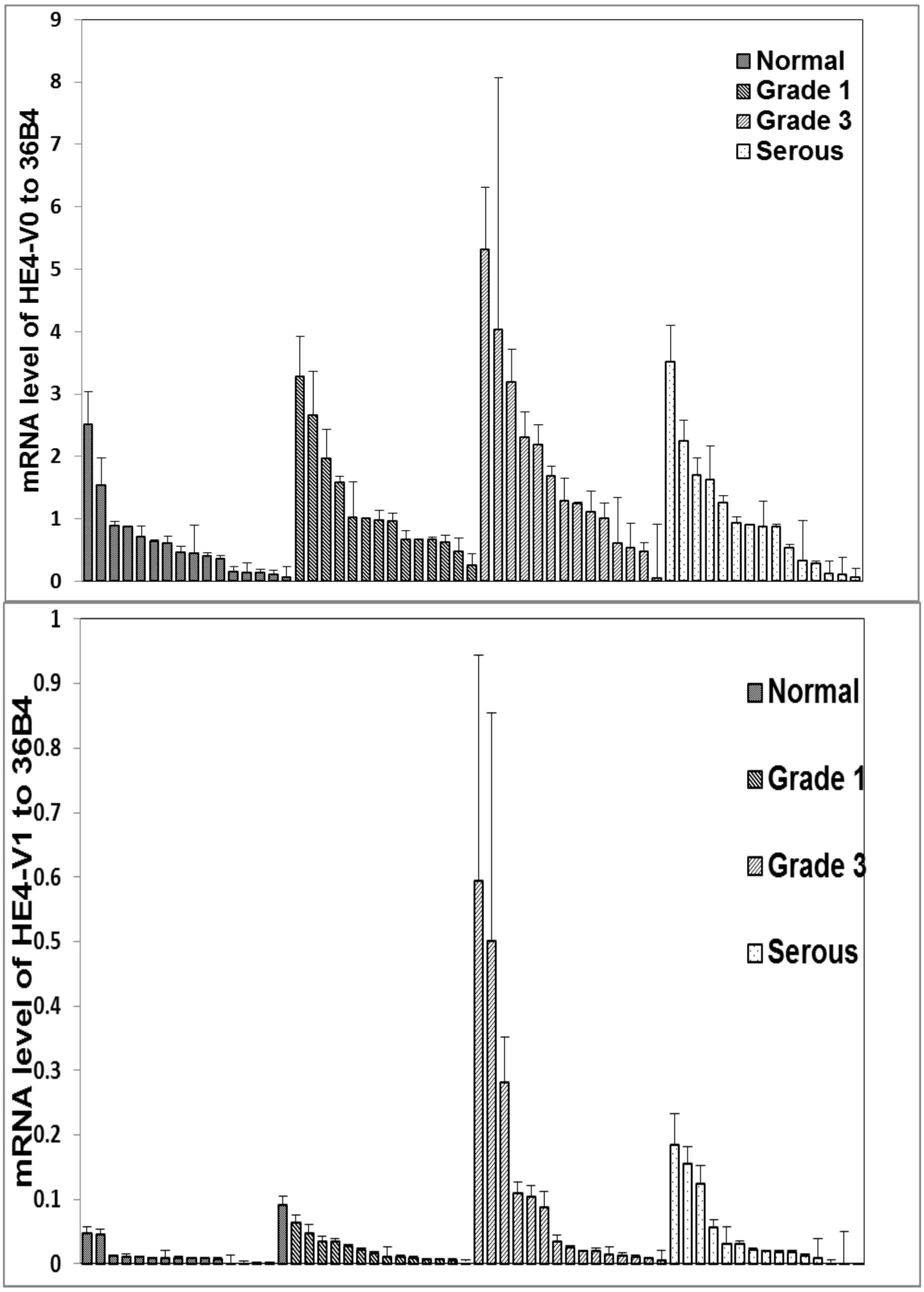

Figure S4. *Cont.*

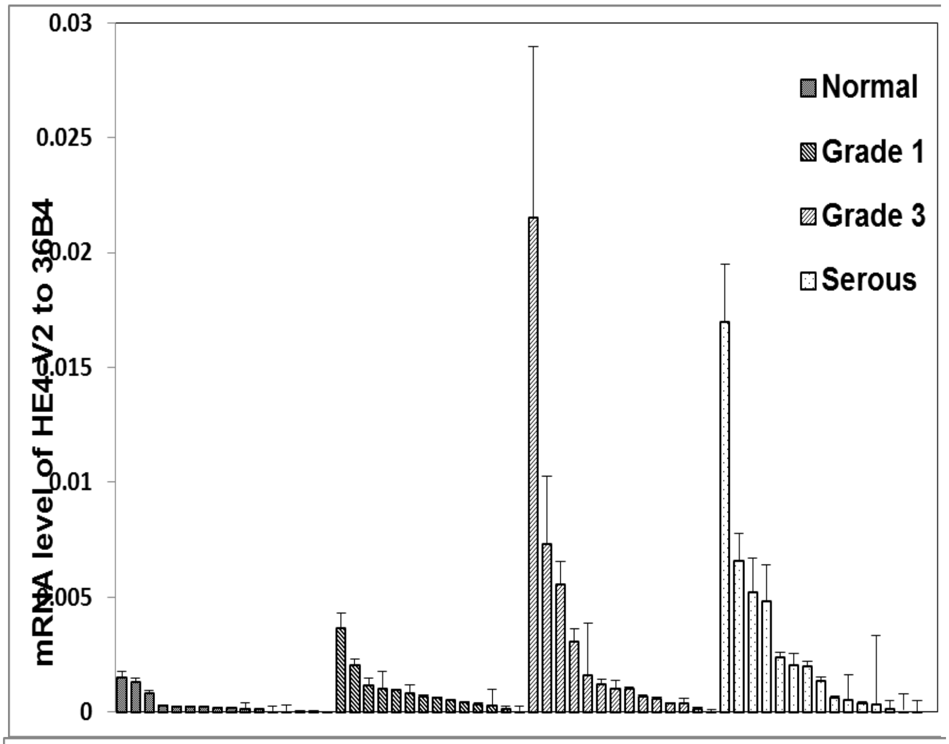

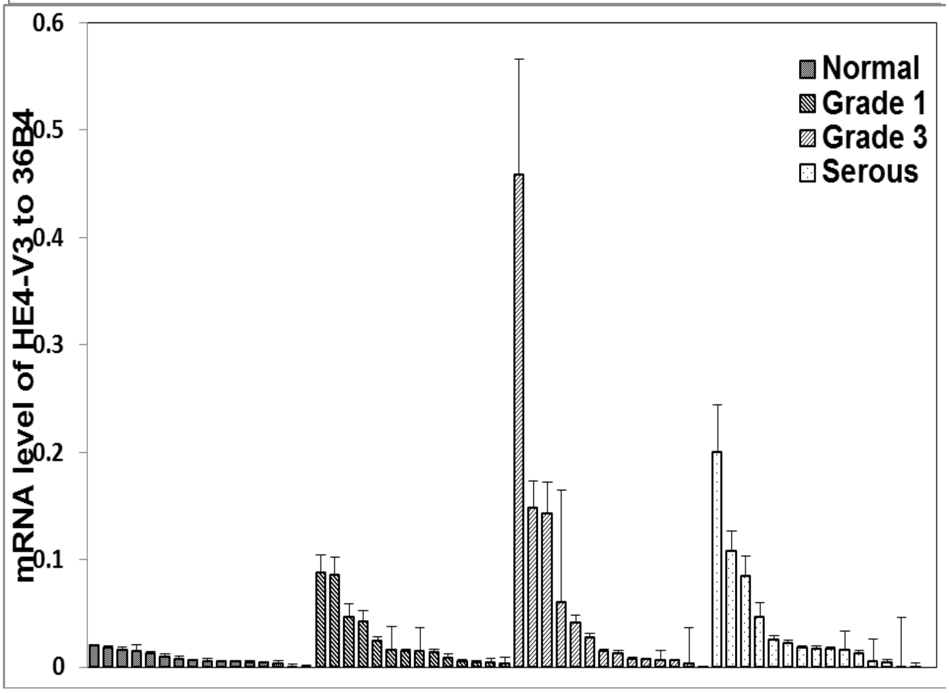

Figure S4. *Cont.*

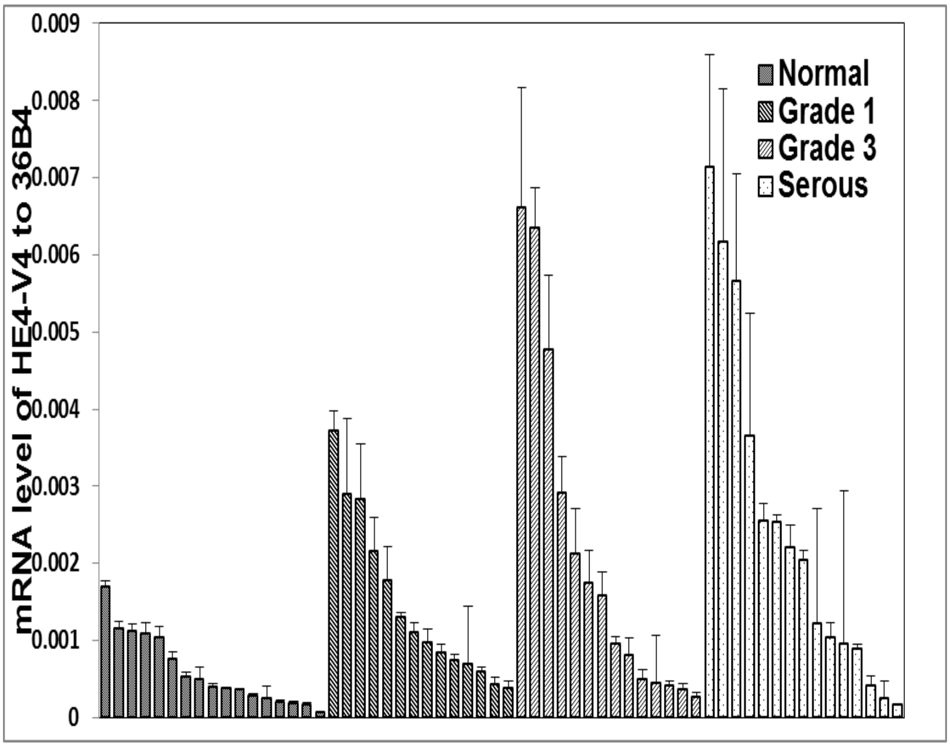

Table S1. Correlation studies on HE4 variant expression pattern in normal endometrium and endometrial cancers. The HE4 variant levels were Log10 transformed and Pearson correlation analysis performed among the normal endometrium, and grade I endometrioid (grade 1) and grade III (grade 3) endometrioid cancers, and papillary serous type EC. There was a statistically significant correlation between each two groups among the three cancer groups, but not between normal endometrium and any cancer groups.

	**Normal**	**Grade I**	**Grade III**	**Serous**	
Normal	1	0.67791<br>0.3221	0.81068<br>0.1893	0.76359<br>0.2364	*r* value<br>*p* value
Grade Ⅰ	0.67791<br>0.3221	1	0.98129<br>0.0031	0.98407<br>0.0024	*r* value<br>*p* value
Grade Ⅲ	0.81068<br>0.1893	0.98129<br>0.0031	1	0.96216<br>0.0088	*r* value<br>*p* value
Serous	0.76359<br>0.2364	0.98407<br>0.0024	0.96216<br>0.0088	1	*r* value<br>*p* value

Table S2. Summary of clinical data on survival, BMI and age of normal and EC specimen. Cell type: NL represents normal epithelium, EM represents endometrioid epithelium, USPC represents uterine serous papillary cell type.

Name	EC cell type	Grade	Age (year)	BMI	Survival (month)
EN1	NL	.	37	23.0469	.
EN2	NL	.	47	35.1393	.
EN3	NL	.	29	25.6312	.
EN4	NL	.	41	29.1749	.
EN5	NL	.	41	25.9089	.
EN6	NL	.	44	39.1347	.
EN7	NL	.	38	24.2447	.
EN8	NL	.	39	27.4756	.
EN9	NL	.	42	26.0417	.
EN10	NL	.	40	40.7407	.
EN11	NL	.	39	37.8329	.
EN12	NL	.	78	26.3486	.
EN13	NL	.	40	37.8138	.
EN14	NL	.	51	27.9543	.
EN15	NL	.	47	32.2528	
EN16	NL	.	41	37.8906	.
IG1	EM	1	49	26.4915	.
IG2	EM	1	52	29.3848	107
IG3	EM	1	55	34.5473	85
IG4	EM	1	51	43.5503	.
IG5	EM	1	52	45.7031	90
IG6	EM	1	49	63.5107	.
IG7	EM	1	75	24.8692	.
IG8	EM	1	57	34.3351	62
IG9	EM	1	64	.	82
IG10	EM	1	86	29.5858	91
IG11	EM	1	39	43.4103	98
IG12	EM	1	62	31.3744	.
IG13	EM	1	62	27.931	85
IG32 SWJ1	EM	2	43	.	.
IIIG1	EM	3	68	19.631	1
IIIG2	EM	3	91	36.1604	41
IIIG3	EM	3	71	34.9277	10
IIIG4	EM	3	59	33.1726	.
IIIG5	EM	3	69	31.2386	180
IIIG6	EM	3	51	28.0428	102
IIIG7	EM	3	53	32.6608	11
IIIG8	EM	3	59	31.1207	65
IIIG9	EM	3	63	44.6163	53

Table S2. *Cont.*

Name	EC cell type	Grade	Age (year)	BMI	Survival (month)
IIIG11	EM	3	77	24.7676	9
IIIG33 SWJ2	EM	3	49	45.8962	64
IIIG3 SWJ4	EM	3	46	28.0816	70
S2	UPSC	3	62	38.9468	27
S3	UPSC	3	64	28.2585	96
S4	UPSC	3	79	29.0249	61
S5	UPSC	3	67	27.5482	8
S6	UPSC	4	50	26.2713	85
S7	UPSC	3	65	25.5593	.
S8	UPSC	3	62	29.685	20
S9	UPSC	4	63	19.433	5
S10	UPSC	3	77	20.9321	1
S11	UPSC	3	59	34.7178	.
S12	UPSC	3	69	34.5271	11
S13	UPSC	3	77	38.9543	13
S14	UPSC	3	93	36.1604	4
S15	UPSC	3	64	33.6485	76
S16	UPSC	3	74	34.375	6

"." Represents data is not available.

© 2013 by the authors; licensee MDPI, Basel, Switzerland. This article is an open access article distributed under the terms and conditions of the Creative Commons Attribution license (http://creativecommons.org/licenses/by/3.0/).
